# Reference intervals for serum cystatin C and serum creatinine in an adult sub-Saharan African population

**DOI:** 10.1186/s12907-019-0086-7

**Published:** 2019-03-18

**Authors:** Bertille Elodie Edinga-Melenge, Adrienne Tchapmi Yakam, Jobert Richie Nansseu, Catherine Bilong, Suzanne Belinga, Eric Minkala, Prisca Armel Noudjeu, Michel Ondhoua, Samuel Walter Kokola, Vicky Joceline Ama Moor, Gloria Ashuntantang

**Affiliations:** 1 Department of Biochemistry, Centre Pasteur of Cameroon, Yaoundé, Cameroon; 2 Department of Physiological Sciences and Biochemistry, Faculty of Medicine and Biomedical Sciences, University of Yaoundé I, Yaoundé, Cameroon; 3 Ebebda District Hospital, Centre Regional Delegation, Ministry of Public Health, Ebebda, Cameroon; 4 Department for the Control of Disease, Epidemics and Pandemics, Ministry of Public Health, Yaoundé, Cameroon; 5 Department of Public Health, Faculty of Medicine and Biomedical Sciences of the University of Yaoundé I, PO Box 1364, Yaoundé, Cameroon; 6 Laboratory of Biochemistry, Yaoundé University Teaching Hospital, Yaoundé, Cameroon; 7 Cardiology and Nephrology Unit, Yaoundé General Hospital, Yaoundé, Cameroon; 8 Department of Internal Medicine and Specialties, Faculty of Medicine and Biomedical Sciences of the University of Yaoundé I, Yaoundé, Cameroon

**Keywords:** Reference interval, Cystatin C, Creatinine, Yaoundé, Cameroon

## Abstract

**Background:**

Serum cystatin C (SCysC) and serum creatinine (SCr) are two biomarkers used in common practice to estimate the glomerular filtration rate (GFR). For SCysC and SCr to be used in a given population, normal values need to be determined to better assess patients. This study aimed to determine SCysC and SCr reference intervals (RIs) in a Cameroonian adult population and factors susceptible of influencing them.

**Methods:**

We carried-out a cross-sectional study from November 2016 to May 2017 in Yaoundé, Cameroon. Participants were Cameroonians aged 18 years and above, residing inside the country and found in good health at study inclusion. SCysC and SCr were determined by particle-enhanced turbidimetric immunoassay standardized against the ERM-DA471/IFCC reference material and by the IDMS reference modified Jaffe kinetic method, respectively. RIs were determined using the 2.5th and 97.5th percentiles and their respective 90% confidence intervals (CIs). The quantile regression served to identify potential factors likely influencing SCysC and SCr values.

**Results:**

We included 381 subjects comprising 49.1% females.. RIs for SCysC varied between 0.57 (90%CI: 0.50–0.60) and 1.03 mg/L (90%CI: 1.00–1.10) for females, and from 0.70 (90%CI: 0.60–0.70) to 1.10 mg/L (90%CI: 1.10–1.20) for males. Concerning SCr, its RIs ranged from 0.58 (90%CI: 0.54–0.61) to 1.08 mg/dL (90%CI: 1.02–1.21) for females, and from 0.74 (90%CI: 0.70–0.80) to 1.36 mg/dL (90%CI: 1.30–1.45) for males. Men had significantly higher SCysC and SCr values than women (*p* <  0.001). Likewise, subjects aged 50 years and above had higher SCysC values in comparison to younger age groups (p <  0.001), which was not the case for SCr values (*p* = 0.491). Moreover, there was a positive and significant correlation between SCysC and SCr in women (ρ = 0.55, *p* < 0.001), in men (ρ = 0.39, *p* < 0.001) and globally (ρ = 0.58; p < 0.001). Furthermore, the sex influenced both biomarkers’ values across all quantile regression models while age and body surface area (BSA) influenced them inconsistently.

**Conclusion:**

This study has determined serum cystatin C and serum creatinine reference intervals in an adult Cameroonian population, whose interpretations might take into account the patient’s sex and to a certain extent, his/her age and/or BSA.

**Electronic supplementary material:**

The online version of this article (10.1186/s12907-019-0086-7) contains supplementary material, which is available to authorized users.

## Background

Glomerular filtration rate (GFR) is widely accepted as the most useful overall index of kidney function in health and disease [1]. It is best evaluated by clearance measurement of exogenous markers such as inuline, but the complex procedures of these measures limit their routine use [2, 3]. GFR is therefore commonly estimated from serum level of endogenous filtration markers. The most widely used and recommended endogenous marker for initial assessment of GFR is serum creatinine [4]. Despite the cheapest cost and the simple use of creatinine-based measurements of GFR, estimation of the level of renal function obtained is quite imprecise. Indeed, the steady-state serum creatinine level is determined by factors that include lean tissue mass; hence, it may vary with sex, age, weight and height [3, 5, 6].

As a result of these limitations, alternative endogenous markers for GFR such as serum cystatin C have been proposed. Cystatin C is a biomarker formed at a constant rate by all nucleated cells of the body which do not correlate with lean tissue mass [5]. Evidence has demonstrated improved accuracy and sensitivity of cystatin C compared to creatinine [7].

For an accurate interpretation of biomarkers levels, reference intervals specific to a population need to be established. Intriguingly and although serum creatinine is widely used in Cameroon, no previous study had yet focused at determining its reference intervals, interpretations relying on western countries’ data. Moreover, considering the growing importance of cystatin C as a prospective marker to assess the renal function, it is obvious that this marker would be introduced in routine clinical practice in Cameroon very soon. Therefore, we conducted the present study to determine the reference values of serum creatinine and cystatin C in a healthy adult Cameroonian population living inside the country. Besides, we aimed to identify potential factors likely influencing these reference intervals.

## Methods

### Study design and setting

This was a population based cross-sectional study conducted between November 2016 and May 2017 in Yaoundé, the capital city of Cameroon. Participants were recruited from the 4 most populated health districts out of the 6 that constitutes the city, namely: *Yaoundé 1, 2, 4 and 6* [8]. Biological analyses were performed at the Centre Pasteur of Cameroun.

### Description of the study population

Participants were adult Cameroonians residing inside the country, aged 18 years and above, found in good health at study inclusion - after a general examination including a brief medical interview, urinalysis and measurement of blood pressure and glycaemia - with no evidence of any acute or chronic illness susceptible of affecting creatinine or cystatin C levels. We excluded known or suspected hypertensives, those with an impaired glucose metabolism (pre-diabetes or diabetes mellitus) or an abnormal dipstick urine test. Pregnant and breastfeeding women were also excluded, as well as drug users. No special dietary recommendations were required. Participants were consecutively recruited during the study period and a minimum of 120 participants was required for each sex group, in line with the International Federation of Clinical Chemistry’s (IFCC) recommendations [9].

### Data collection

Participants were mostly recruited in churches, schools/universities/colleges and mosques. On the days of recruitment, each potential participant was required to sign a consent form as the testimony of his/her volunteering participation. Subsequently, he/she underwent a brief interview using a preconceived, standardized and pre-tested questionnaire (Additional file 1); then a summary physical examination was conducted, during which blood pressure was measured. We used the simplified calculation procedure from Mosteller RD to derive each participant’s body surface area (BSA)) [10]. In addition, a urine sample was collected for dipstick urine analysis and a capillary glycaemia was performed using a OneTouch® analyzer.

### Biochemical assays

Ten milliliters of venous blood were collected by venipuncture in 2 dry tubes of 5 ml each. Serum was separated by centrifugation at 3000 rpm within 10 min. Biochemical assays were conducted using the autoanalyzer Cobas C 501/6000, Roche Diagnostics, USA. Serum cystatin C was measured in increments of 0 .1mg/L by particle-enhanced turbidimetric immunoassay using Tina-quant® Cystatin C reagent kits (Roche Diagnostics, USA). The method applied was standardized against the ERM-DA471/IFCC reference material. Meanwhile, serum creatinine was determined by the Isotope Dilution Mass Spectrometry (IDMS) reference modified Jaffe kinetic method using Creatinine Jaffe Cobas® reagent kits (Roche Diagnostics, USA).

### Statistical analysis

Data were coded and entered using the Census and Survey Processing System version 7.1. Statistical analysis was performed using the Statistical Package for Social Sciences version 23.0 (IBM SPSS Inc., Chicago, Illinois, USA) and STATA version 12.0 (STATACORP, Texas, USA). Categorical variables are presented using frequency (percentage) while continuous variables are summarized with their median [interquartile range, IQR]. The Kolmogorov Smirnov test was used to assess the normality of continuous variables’ distributions. Reference intervals (RIs) were determined by the nonparametric method as described in the IFCC guidelines [11]. This method was used to determine the 2.5 and 97.5 percentiles and the respective 90% confidence intervals (CI) around these estimates. The Mann–Whitney U-test and the Kruskal-Wallis H-test were used for bivariate analyses, to compare the distributions of continuous variables, considering that these variables did not follow a Gaussian shape. For the same reason, it is the Spearman correlation test (with its rho (ρ) coefficient) that was used to investigate existence of any correlation between continuous variables including serum cystatin C, serum creatinine and age. Furthermore, we used a 25th, 50th and 75th percentile quantile regression analysis to identify any factor likely influencing serum cystatin C or serum creatinine reference intervals in a model including the age, sex, and BSA. Statistical significance was set at a *p*-value lower than 0.05.

## Results

A total of 485 healthy subjects were screened of whom 104 were excluded because of underlying diabetes mellitus, pre-diabetes, hypertension or abnormal dipstick urine test. The reference population comprised 381 healthy adults (including 49.1% females) aged between 18 and 71 years old with a median age of 28 years [IQR 23–40]. There were no differences in the distribution of age between male and female participants (*p* = 0.290). By contrast, males had significantly higher BSA values than females: *p* = 0.002(Table 1).

**Table 1:** Reference intervals for serum cystatin C and serum creatinine according to sex

Parameter	All (*n* = 381)	Males (*n* = 194)	Females (*n* = 187)	p*
Age (years)	28 [23–40]	28 [24–40]	26 [22–43]	0.290
BSA (m^2^)	1.68 [1.55–1.79]	1.70 [1.81]	1.65 [1.52–1.76]	0.002^*^
Serum cystatin C (mg/L)				
Median [IQR]	0.80 [0.70–0.90]	0.90 [0.80–1.00]	0.80 [0.70–0.90]	< 0.001
2.5th percentile (90%CI)	0.60 (0.60–0.61)	0.70 (0.60–0.70)	0.57 (0.50–0.60)	
97.5th percentile (90%CI)	1.10 (1.10–1.11)	1.10 (1.10–1.20)	1.03 (1.00–1.10)	
Serum creatinine (mg/dL)				
Median [IQR]	0.92 [0.77–1.06]	1.06 [0.96–1.14]	0.79 [0.71–0.88]	< 0.001
2.5th percentile (90% CI)	0.61 (0.59–0.64)	0.74 (0.70–0.80)	0.58 (0.54–0.61)	
97.5th percentile (90% CI)	1.30 (1.28–1.35)	1.36 (1.30–1.45)	1.08 (1.02–1.21)	

*BSA* body surface area, *CI* confidence interval, *IQR* interquartile range, *SCysC* serum cystatin C, *SCr* serum creatinine; ^†^The Mann-Whitney U-test was used for variable comparisons; ^*^p < 0.05

The non-parametric reference intervals for serum cystatin C were 0.57–1.03 mg/L for women and 0.70–1.10 mg/L for men; the reference intervals for the whole study population were 0.60–1.10 mg/L (Table 1). For serum creatinine, these intervals were 0.58–1.08 mg/dL for women, 0.74–1.36 mg/dL for men, and 0.61–1.30 mg/dL for all subjects (Table 1). As compared to women, men had significantly higher titers of serum cystatin C (median 0.90 vs. 0.80 mg/L; *p* < 0.001; Table 1) than women, except for those aged 50 years and above (*p* = 0.125; Table 2). Similarly, men had significantly higher serum creatinine values (median 1.06 vs. 0.79 mg/dL; *p* < 0.001; Table 1) than women, this tendency being the same in all age groups (Table 3).

**Table 2:** Reference intervals for serum cystatin C by age and sex

Age (years)	*N* = 381	Serum cystatin C (mg/L)			
		Median (IQR)	2.5th percentile (90%CI)	97.5th percentile (90%CI)	p^┼^
< 20	Male: 9	0.9 (0.85–1.05)	0.7 (0.70–0.90)	1.1	0.003
	Female: 14	0.75 (0.70–0.80)	0.6 (0.60–0.70)	0.9	
	All: 23	0.8 (0.70–0.90)	0.6 (0.60–0.70)	1.1	
[20–30]	Male: 95	0.9 (0.80–1.0)	0.6 (0.60–0.70)	1.1 (1.10–1.10)	< 0.001
	Female: 89	0.7 (0.70–0.80)	0.6 (0.50–0.60)	1.0 (0.90–1.00)	
	All: 184	0.8 (0.70–0.90)	0.6 (0.60–0.60)	1.1 (1.10–1.10)	
[30–40]	Male: 40	0.9 (0.80–0.90)	0.7 (0.70–0.80)	1.0 (1.00–1.00)	0.001
	Female: 31	0.8 (0.60–0.80)	0.5 (0.50–0.50)	1.0	
	All: 71	0.8 (0.80–0.90)	0.5 (0.50–0.58)	1.0 (1.00–1.00)	
[40–50]	Male: 32	0.9 (0.80–0.98)	0.7 (0.70–0.70)	1.1	0.002
	Female: 21	0.8 (0.70–0.90)	0.6 (0.60–0.70)	1.0	
	All: 53	0.8 (0.80–0.90)	0.6 (0.60–0.70)	1.0 (1.00–1.00)	
≥ 50	Male: 18	1.0 (0.88–1.0)	0.7 (0.70–0.80)	1.2	0.125
	Female: 32	0.9 (0.80–0.90)	0.6 (0.60–0.80)	1.1	
	All: 50	0.9 (0.80–1.00)	0.7 (0.60–0.73)	1.2 (1.10–1.20)	

*CI* confidence interval, *IQR* interquartile range. Some 90% confidence intervals are not presented due to the small number of participants in corresponding age groups. ^┼^ The Mann-Whitney U-test was used to compare the distribution of serum cystatin C values between males and females. The difference between age-groups was significant when using the Kruskal-Wallis H-test (*p* < 0.001)

**Table 3:** Reference intervals for serum creatinine by age and sex

Age (years)	*N* = 381	Serum creatinine (mg/dL)			
		Median (IQR)	2.5th percentile (90%CI)	97.5th percentile (90%CI)	p^┼^
< 20	Male: 9	1.09 (1.00–1.15)	0.80 (0.80–1.04)	1.18	< 0.001
	Female: 14	0.76 (0.72–0.79)	0.66 (0.66–0.70)	0.88	
	All: 23	0.79 (0.75–1.08)	0.66 (0.66–0.70)	1.18	
[20–30]	Male: 95	1.03 (0.94–1.12)	0.80 (0.70–0.85)	1.30 (1.29–1.35)	< 0.001
	Female: 89	0.79 (0.71–0.87)	0.60 (0.54–0.61)	1.07 (1.01–1.28)	
	All: 184	0.91 (0.78–1.05)	0.61 (0.59–0.65)	1.30 (1.26–1.33)	
[30–40]	Male: 40	1.06 (0.95–1.13)	0.75 (0.69–0.81)	1.29	< 0.001
	Female: 31	0.73 (0.65–0.83)	0.57 (0.57–0.60)	1.26	
	All: 71	0.94 (0.74–1.08)	0.58 (0.57–0.62)	1.27 (1.22–1.32)	
[40–50]	Male: 32	1.08 (0.99–1.17)	0.68 (0.68–0.74)	1.45	< 0.001
	Female: 21	0.74 (0.69–0.90)	0.51 (0.51–0.66)	1.02	
	All: 53	0.99 (0.74–1.10)	0.61 (0.51–0.66)	1.38 (1.24–1.45)	
≥ 50	Male: 18	1.17 (0.99–1.30)	0.79 (0.79–0.96)	1.52	< 0.001
	Female: 32	0.85 (0.79–0.93)	0.60 (0.60–0.65)	1.05	
	All: 50	0.93 (0.81–1.06)	0.64 (0.60–0.66)	1.43 (1.33–1.52)	

*CI* confidence interval, *IQR* interquartile range. Some 90% confidence intervals are not presented due to the small number of participants in corresponding age groups. ^┼^ The Mann-Whitney U-test was used to compare males and females; the difference in the distribution of serum creatinine values between age-groups was not significant with the Kruskal-Wallis H-test (*p* = 0.491)

Additionally, serum cystatin C levels were higher in persons aged 50 years and above compared to their counterparts aged less than 50 years old (p < 0.001; Table 2); on the contrary, this difference was not observed with serum creatinine values (*p* = 0.491; Table 3). Moreover, we found a positive and significant correlation between serum cystatin C and serum creatinine both in females (ρ = 0.55, *p* < 0.001), in males (ρ = 0.39, p < 0.001) and in the total study population (ρ = 0.58; *p* < 0.001).

**Fig. 1:**
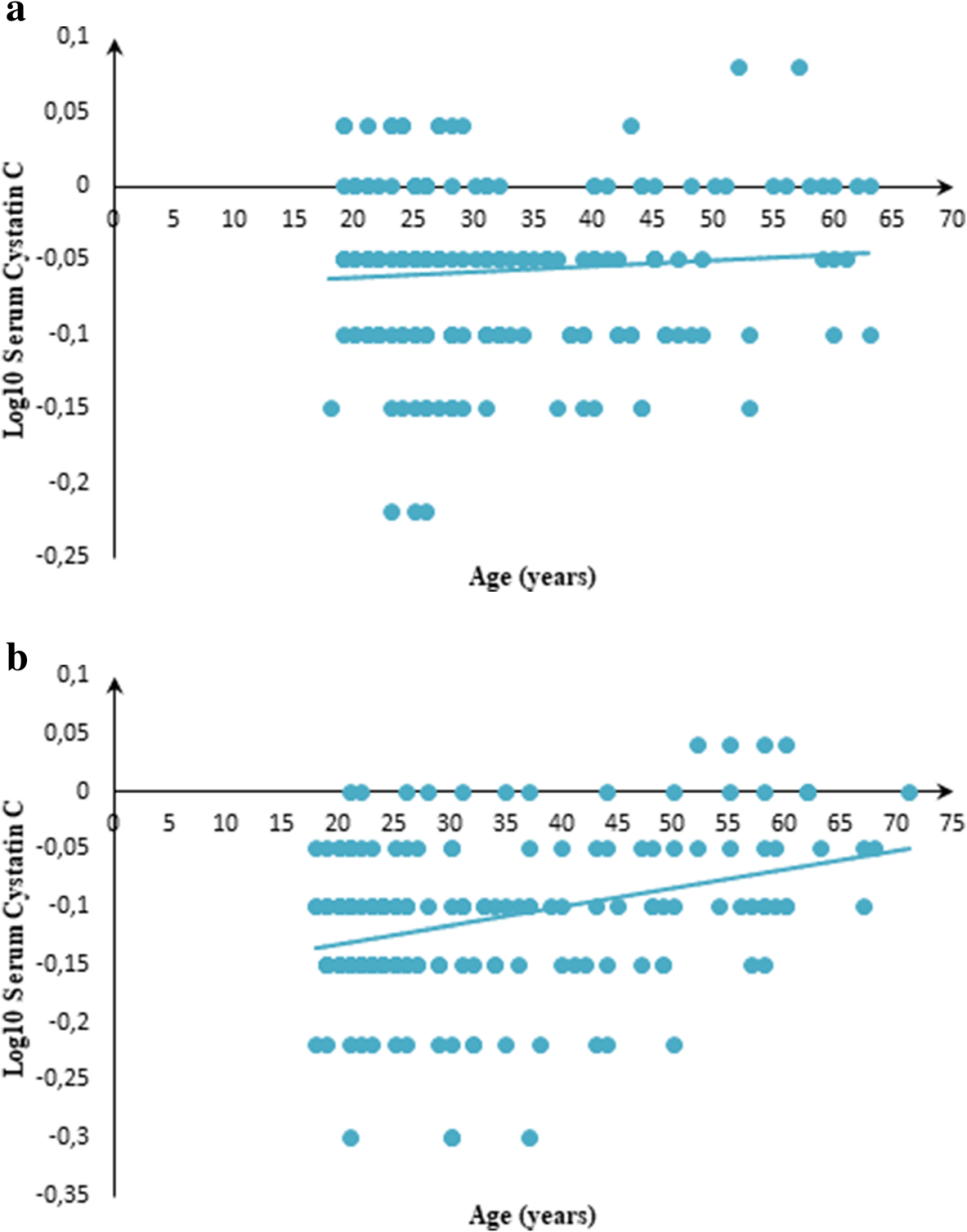
**a** Relationship between serum cystatin C (log) values and age in males [(*n* = 194); y = 0.0009x – 0.0114, ρ = 0.162 (*p* = 0.024)]. **b** Relationship between serum cystatin C (log) values and age in females [(*n* = 187); y = 0.0016x – 0.164, ρ = 0.265 (*p* < 0.001)]

**Fig. 2:**
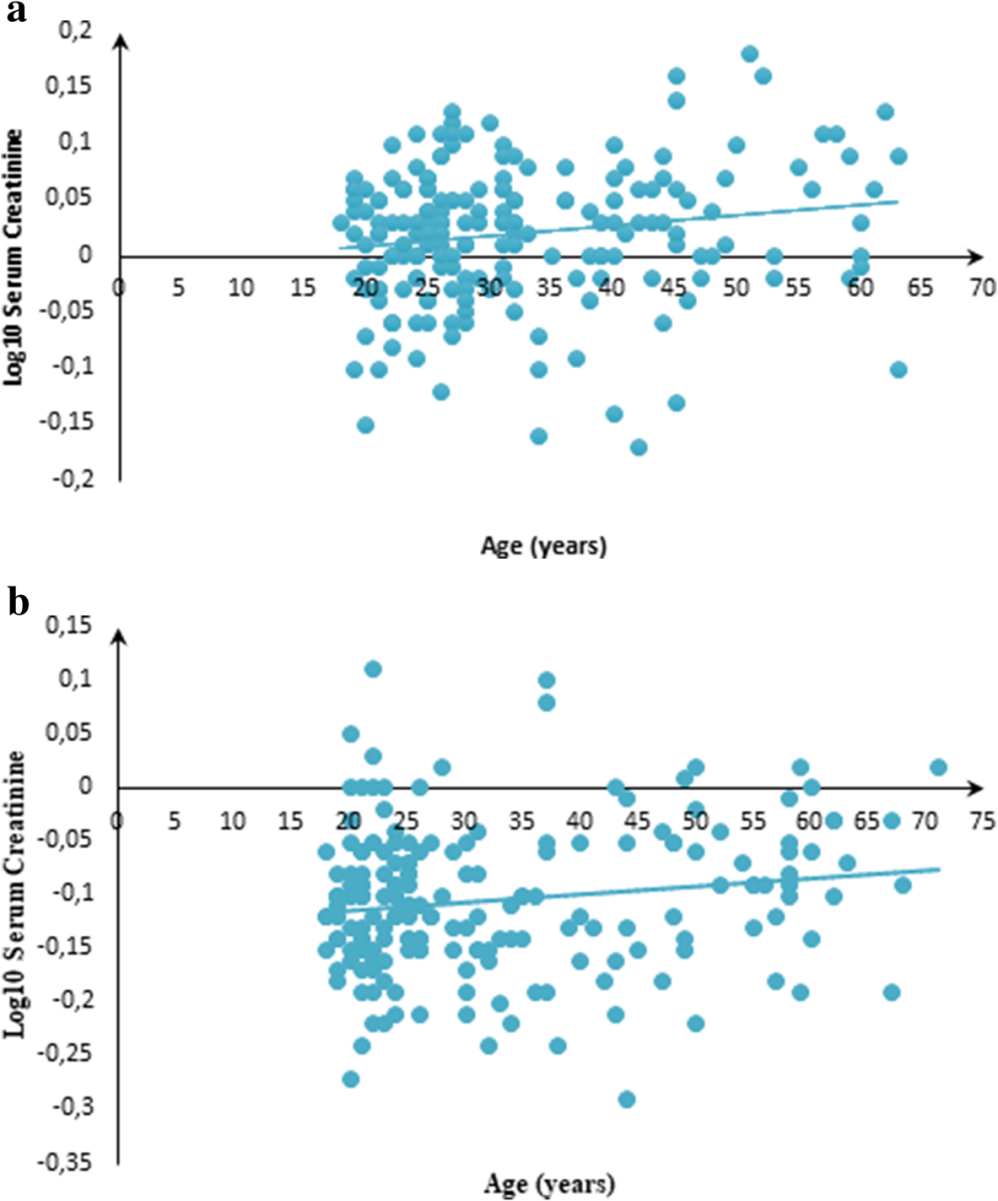
**a** Relationship between serum creatinine (log) values and age in males [(n = 194); y = 0.0009x – 0.0114, ρ = 0.162 (*p* = 0.024)]. **b** Relationship between serum creatinine (log) values and age in females [(n = 187); y = 0.0007x – 0.129, ρ = 0.127 (*p* = 0.082)]

Furthermore, the correlation between serum cystatin C logarithmically-transformed values and age was weak and non-significant in males (ρ = − 0.006, *p* = 0.930; Fig. 1 a), but became significant in females (ρ = 0.265, *p* < 0.001; Fig. 1 b. Contrariwise, the correlation between serum creatinine logarithmically-transformed values and age was significant in males (ρ = 0.162, *p* = 0.024; Fig. 2 a), but insignificant in females (ρ = 0.127, *p* = 0.082; Fig. 2 b). On the other hand, results of the quantile regression which are presented in Table 4 showed that across the various models, the sex remained the only factor likely influencing both serum cystatin C and serum creatinine values. The age seemed to contribute in explaining serum cystatin C values in the 75th percentile quantile regression model, which was identical for serum creatinine values. The BSA was contributive in explaining serum creatinine values only in the 50th percentile quantile regression model (Table 4).

**Table 4:** Regression coefficients and *p*-values for the 25th, 50th and 75th percentiles quantile regression models

	Serum cystatin C			Serum creatinine		
	25th	50th	75th	25th	50th	75th
Sex	−0.1 (< 0.001)*	− 0.1 (< 0.001)*	−0.09 (< 0.001)*	−0.24 (< 0.001)*	−0.28 (< 0.001)*	−0.27 (< 0.001)*
Age	7.67e^−19^ (1.000)	− 3.36e^− 18^ (1.000)	0.003 (0.001)*	0.0007 (0.171)	0.001 (0.162)	0.002 (0.031)*
BSA	9.39e^−17^ (1.000)	7.35e^−16^ (1.000)	0.038 (0.475)	0.08 (0.072)	0.15 (0.001)*	0.127 (0.073)

*p < 0.05

## Discussion

In agreement with IFCC recommendations [11], the reference intervals for serum cystatin C and serum creatinine were determined in the present study among a healthy Cameroonian adult population. Our results revealed that the reference intervals for serum cystatin C varied between 0.6 and 1.1 mg/L, with men having higher values than women (*p* < 0.001), except in the 50+ years age group. Concerning serum creatinine, the reference intervals ranged from 0.6 to 1.3 mg/dL; similarly, men had significantly higher levels than women (*p* < 0.001) across all age groups. Participants aged 50 years and above had higher serum cystatin C values than those aged less than 50 years (*p* < 0.001), which was not the case for serum creatinine values (*p* = 0.491). Moreover, the correlation between serum cystatin C and serum creatinine was positive and significant (ρ = 0.58; *p* < 0.001) and the quantile regression pointed mostly the sex, and to a certain extent the age and BSA as independent factors susceptible of influencing serum cystatin C and/or serum creatinine values.

Reference intervals for serum cystatin C obtained in this study (0.60–1.10 mg/L) are in compliance with those from previous studies which have also used turbidimetric assay. For instance, Köttgen et al. recorded in a US population a reference interval varying between 0.61–1.04 mg/L; Okonkwo et al. in a Nigerian population recorded a reference interval ranging between 0.64–1.12 mg/L and Li et al. in a Chinese population recorded a reference interval varying from 0.60 to 1.08 mg/L [12–14]. By contrast, the reference intervals for serum creatinine obtained in this study (0.61–1.3 mg/dL) seem to differ from that of Caucasians. Indeed, Pottel et al. found reference intervals around 0.48–0.93 mg/dL in women and 0.63–1.16 mg/dL in men within a healthy adult Caucasian population [15]. These intervals concur with those of Ceriotti et al. obtained in a multicenter analysis of three studies based on Caucasian adults. In this study indeed, the reference intervals for serum creatinine varied between 0.45–0.92 mg/dL in women and 0.59–1.05 mg/dL in men [16]. These differences could be explained by the fact that the measurement of serum creatinine used enzymatic methods in the two studies just cited, which could give slightly lower values than colorimetric assays that were used in our study. Additionally, evidence has accumulated that black people have a more important lean tissue mass and a lower GFR compared to Caucasians [3, 17]. However, our results corroborate those from other African authors such as Sakande et al. in Burkina Faso and Dosoo et al. in Ghana. Indeed, Sakande et al. reported reference intervals ranging between 0.63–1.41 mg/dL in men and 0.45–1.24 mg/dL in women; reference intervals obtained by Dosoo et al. were 0.63–1.35 mg/dL in men and 0.60–1.20 mg/dL in women [18, 19]. Furthermore, Lim et al. conducted a study among afro-Americans and found similar results with men having serum creatinine reference intervals around 0.73–1.45 mg/dL and women, around 0.52–1.15 mg/dL [20].

The sex-related differences in the non-parametric reference intervals for serum creatinine are in line with previous studies and reinforced by results of our quantile regression analysis indicating that the sex influenced serum creatinine values across all models, while adjusting for age and BSA. Indeed, muscular mass is higher in men compared to women [3, 5, 6, 21]. Concurring with previous findings, our results indicate that serum cystatin C levels seem to be slightly affected by factors such as sex and age [22–24]. Pottel et al. showed for instance that cystatin C increases with age, after the age of 70 years old [24]. The influence of sex on serum cystatin C levels is still unclear. In fact, some studies have reported that serum cystatin C levels are independent of sex unlike other studies have claimed that sex influences significantly serum cystatin C values [13, 23, 25–28].

In our study for instance, we found that the sex constituted one independent explanatory factor for serum cystatin C values, whatever the quantile regression model considered; additionally, serum cystatin C levels were 11% higher in men than in women (0.90 mg/L vs 0.80 mg/L; *p* < 0.001). These results corroborate those from Köttgen et al. in the US who reported a difference of 8% between males and females [12]. However, Al Wakeel et al. in a Saudi adult population reported lower serum cystatin C levels in men compared to women (0.72 mg/L vs 0.77 mg/L; p < 0.001) as well as Li et al. in China (0.84 mg/L vs 0.85 mg/L; *p* < 0.05) [14, 29]. In the Saudi study, women had higher body mass index that men and the positive correlation between serum cystatin C and body mass index could have explained the higher serum cystatin C levels in women [13, 29, 30]. In Li et al.’s study, the sex difference was observed only between 30 and 60 years [14].

Likewise, we found in our study that from 50 years old and beyond, differences of serum cystatin C levels between men and women became non-significant (median 1.00 vs 0.90 mg/L; *p* = 0.125) while the difference persisted for serum creatinine levels (median 1.17 vs 0.85 mg/dL; p < 0.001). Actually, the influence of sex on serum cystatin C levels seems non-significant with increasing age, suggesting a physiological or pathological condition which should be more investigated in elderly. Further studies are warranted in this respect.

On the other hand, subjects aged 50 years and over had 11% higher serum cystatin C levels compared to lower age groups (0.90 vs. 0.80; *p* < 0.001). Concurring with these results, several other studies have demonstrated an increase in cystatin C values above a threshold age varying from 40 to 70 years [12, 14, 24, 29–32]. The higher levels of serum cystatin C in older subjects could be due to the physiological decrease in GFR which starts from 40 years [33].

Serum creatinine levels are also expected to increase around the same age (≥50 years); however, we observed that the distributions of serum creatinine values were similar across the various age groups (*p* = 0.491). Likewise, Pottel et al. using a Caucasian population noticed that between 20 and 70 years old, the mean serum creatinine level was stable [24]. This could be explained by the drop in creatinine rate production due to reduction in the muscle mass which appears concomitantly with the decrease in GFR [33]. The physiological increase in creatinine levels will be therefore lately observed around 65–70 years [3, 15].

We found a positive and significant correlation between serum cystatin C and serum creatinine, both in males (ρ = 0.39, *p* < 0.001), in females (ρ = 0.55, p < 0.001) and in the total population (ρ = 0.58; *p* < 0.001). These findings mirror those from Pottel et al. who reported a positive correlation between these two biomarkers in a Caucasian population of 8584 subjects (r = 0.87; *p* < 0.0001) [34]. Potter et al.’s correlation coefficient was higher than ours, perhaps because they used the Pearson correlation test and rescaled their biomarkers.

The inconsistent influence of age on both serum cystatin C and serum creatinine values was observed after applying the quantile regression analysis. Indeed, we found that age influenced significantly both serum cystatin C and serum creatinine values only at the 75th percentile quantile regression model, the estimator being insignificant at the 25th and 50th percentile models. We need further well-designed studies to better investigate the influence of age (and BSA) on serum cystatin C and serum creatinine values in our context.

However, our findings need to be interpreted in the context of some limitations, mainly occurring from the non-random sampling method used and single measurement of serum cystatin C and serum creatinine. In fact, the representativeness of our study population and generalization of our results to the entire Cameroonian population would have been better obtained with randomization. Nevertheless, we selected the most populated health districts among the 6 that compose Yaoundé, the cosmopolitan capital city of Cameroon. On the other hand, participants were selected on the basis of their normal renal function which could be attested only by measurement of GFR by the gold standard (inuline). Nonetheless, the absence of risk factors for kidney disease and the normal clinical and biological tests performed among our participants could be some indirect indicators of normal kidney function. Furthermore, we used rigorous statistical procedures and applied the IFCC guidelines to depict our estimates. Notwithstanding and to the very best of our knowledge, this is the first study providing the reference values for serum cystatin C and serum creatinine in Cameroon, which could be translatable to similar sub-Saharan African populations.

## Conclusion

This study depicted serum cystatin C and serum creatinine reference intervals in a healthy adult Cameroonian population. Men had significantly higher levels of both biomarkers compared to women. Subjects aged 50 years old and above had significantly higher serum cystatin C values than those aged less than 50 years old. Therefore, the interpretation of both biomarkers should probably take into account the patient’s sex and to a certain extent, his/her age (and/or body surface area) for an appropriate diagnosis of a renal disease. Moreover, it is hoped that our data stimulate further research on a larger population that will be more representative of the whole country’s diversity.

## Additional file

Additional file 1: Questionnaire. (DOCX 17 kb)

## Abbreviations

BSA: Body surface area
CI: Confidence interval
GFR: Glomerular filtration rate
IFCC: International Federation of Clinical Chemistry
IQR: Interquartile range
RI: Reference interval
SCr: Serum creatinine
SCysC: Serum cystatin C
