# Supplementary material for: Reference intervals for serum cystatin C and serum creatinine in an adult sub-Saharan African population
Source: BMC Clin Pathol. 2019 Mar 18;19:4. doi: 10.1186/s12907-019-0086-7 (PMC6423796; doi:10.1186/s12907-019-0086-7)
Supplement: Supplementary file 1 — Questionnaire. (DOCX 17 kb) [file 12907_2019_86_MOESM1_ESM.docx]

## Questionnaire

**Section 1 : Identification and sociodemographic characteristics**

| S1Q01 | Participant code | \|__\|__\|/__\|__\|/__\|__\| | | |
| --- | --- | --- | --- | --- |
| S1Q02 | Gender | Male = 1  Female = 2 | \|__\| | |
| S1Q03 | Date of birth | \|__\|__\|/__\|__\|/__\|__\| | | |
| S1Q04 | Ethnic group | Bantu = 1  Semi-bantu = 2  Soudanese= 3 | | \|__\| |

**Section 2: Clinical and anthropometric parameters**

| S2Q01 | Weight (Kg) | \|__\|__\|__\| |
| --- | --- | --- |
| S2Q02 | Height (m) | \|__\|,\|__\|__\| |
| S2Q03 | BMI (Kg/m^2^) | \|__\|__\|,\|__\| |
| S2Q04 | Waist circumference (cm) | \|__\|__\|__\| |

**Section 3: Biological testings**

|  | **Laboratory assay** | **Result** |
| --- | --- | --- |
| S3Q01 | Serum Cystatin C (mg/L) | \|__\|__\|,\|__\|__\| |
| S3Q02 | Serum Creatinine (mg/dL) | \|__\|__\|,\|__\|__\| |
